# Monitoring of dynamic changes in Keyhole Limpet Hemocyanin (KLH)-specific B cells in KLH-vaccinated cancer patients

**DOI:** 10.1038/srep43486

**Published:** 2017-03-07

**Authors:** Florian Wimmers, Nienke de Haas, Anja Scholzen, Gerty Schreibelt, Elles Simonetti, Marc J. Eleveld, Huberdina M. L. M. Brouwers, Marjo Beldhuis-Valkis, Irma Joosten, Marien I. de Jonge, Winald R. Gerritsen, I. Jolanda M. de Vries, Dimitri A. Diavatopoulos, Joannes F. M. Jacobs

**Affiliations:** 1 Department of Tumor Immunology, Radboud Institute for Molecular Life Sciences, Radboud university medical center, Nijmegen, The Netherlands; 2 Department of Medical Microbiology, Radboud university medical center, Nijmegen, The Netherlands; 3 Department of Pediatrics, Laboratory of Pediatric Infectious Diseases, Radboud university medical center, Nijmegen, The Netherlands; 4 Department of Laboratory Medicine, Laboratory Medical Immunology, Radboud university medical center, Nijmegen, The Netherlands; 5 Department of Medical Oncology, Radboud university medical center, Nijmegen, The Netherlands

## Abstract

Keyhole limpet hemocyanin (KLH) is used as an immunogenic neo-antigen for various clinical applications and during vaccine development. For advanced monitoring of KLH-based interventions, we developed a flow cytometry-based assay for the *ex vivo* detection, phenotyping and isolation of KLH-specific B cells. As proof-of-principle, we analyzed 10 melanoma patients exposed to KLH during anti-cancer dendritic cell vaccination. Our assay demonstrated sensitive and specific detection of KLH-specific B cells in peripheral blood and KLH-specific B cell frequencies strongly correlated with anti-KLH serum antibody titers. Profiling of B cell subsets over the vaccination course revealed that KLH-specific B cells matured from naïve to class-switched memory B cells, confirming the prototypic B cell response to a neo-antigen. We conclude that flow-cytometric detection and in-depth phenotyping of KLH-specific B cells is specific, sensitive, and scalable. Our findings provide novel opportunities to monitor KLH-specific immune responses and serve as a blueprint for the development of new flow-cytometric protocols.

Keyhole limpet hemocyanin (KLH) is a high-molecular-weight glycoprotein of marine origin that induces both cell-mediated and humoral responses in animals and humans. Because of its potent immunogenicity, its low-grade toxicity and its availability as a clinical grade product, KLH is used extensively as a natural immunostimulant for basic research and clinical applications^1^,^2^,^3^. As a neo-antigen, KLH is ideally suited to study T cell-dependent primary and secondary immune responses and a recent study highlights its ability to stimulate the innate immune system. KLH was first introduced into the clinic in 1967 to assess immunocompetence of individuals^4^. KLH is currently mainly employed as standard carrier protein for the production of monoclonal antibodies to haptens such as peptides and oligosaccharides^1^. Besides this, KLH has been studied as a local treatment for patients with bladder cancer, but proved to be inferior to mitomycin treatment^5^,^6^. Finally, KLH has progressed into clinical trials as either a carrier protein, an adjuvant- or immunomonitoring tool in a variety of cancer vaccines^7^,^8^ and immunotherapeutic strategies against chronic infections and autoimmune disease^9^,^10^. Strong inter-individual differences are typically observed in the immunological and clinical responses of individuals exposed to KLH^8^. In-depth information about the dynamics and phenotype of the KLH-specific immune response may help to optimize its clinical use and provide biomarkers for selecting patients that will benefit most from KLH-based interventions.

We currently lack appropriate monitoring tools that allow a detailed study of the KLH-specific B cell response. So far, B cell responses to KLH have mainly been evaluated by quantifying KLH-specific antibodies in serum^11^,^12^,^13^,^14^,^15^,^16^. Direct longitudinal analysis of KLH-specific B cells in peripheral blood could provide novel information on the magnitude and phenotype of the KLH-specific B cell response. A number of recent studies employed fluorescently-labeled antigens to directly monitor vaccine-, virus- or allergen-induced antigen-specific B cells^17^,^18^,^19^,^20^.

In this study, we established a novel flow-cytometric assay to detect, phenotype and isolate KLH-specific B cells in peripheral blood in a sensitive and specific manner. As proof of concept, we applied our novel assay to monitor KLH-specific B cell responses in a cohort of cancer patients that were vaccinated with autologous monocyte-derived matured dendritic cells (DC) loaded with KLH and tumor antigen. We found that the serum concentration of KLH-specific antibodies was highly correlated to the number and phenotype of KLH-specific B cells. Flow-cytometric isolation of the fluorescently labeled KLH-specific B cells allowed *ex vivo* production of KLH-specific antibodies and confirmed the high specificity of the assay. By analyzing B cell maturation, we were able to visualize the dynamics of KLH-specific B cells following primary as well as booster vaccination.

Our novel assay allows detailed cellular monitoring of the KLH-specific B cell response. Applying this technique to the field of KLH-based interventions could provide new insight into the origin, development and maintenance of the KLH-specific response and may facilitate the development of novel KLH-applications.

## Results

To gain an understanding of the B cell response to KLH, we set out to examine the frequency and phenotype of KLH-specific B cells across the DC vaccination course of 10 stage III melanoma patients (Supplementary Table 1). To cover multiple stages of humoral immunity, we selected three time points during treatment to measure the primary response as well as the recall response within each patient. To examine the primary response, baseline frequencies were determined 7–22 days before vaccination and after injection number 2–4 of the 1^st^ cycle (designated 1^st^ cycle). Recall responses were determined after 3 injections of the 3^rd^ vaccination cycle (designated 3^rd^ cycle).

### Detection of KLH-specific B cells via flow cytometry is specific and sensitive

**Figure 1:**
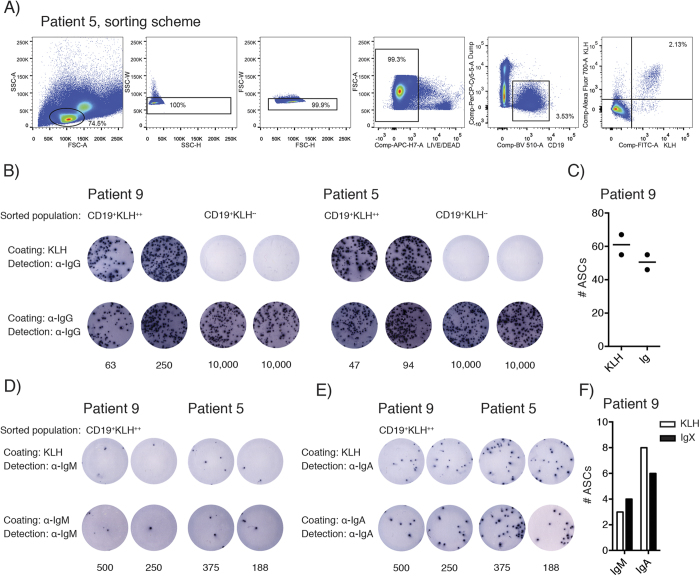
KLH^++^ B cells produce antibodies that are specific for KLH. (**A**) KLH^++^ and KLH^−−^ CD19^+^ B cells from patient 5 and patient 9 were FACS sorted and differentiated into antibody secreting cells (ASCs). Plates were coated with either KLH or with anti-IgG, IgM or IgA, after which differentiated KLH^++^ and KLH^−−^ B cells were added to the wells. Subsequently, ASCs secreting anti-KLH and anti-total IgG- (**B**), anti-KLH and anti-total IgM- (**D**) and anti-KLH and anti-total IgA-antibodies (**E**) were detected using ELISPOT. For patient 9, ASCs were enumerated and compared between specific and unspecific conditions (C, F white bars indicate KLH-specific ASCs, black bars total ASCs). The number of sorted and *ex vivo* differentiated cells is indicated below ELISPOT photographs. (**B,D,E**) For each condition two technical replicates with different numbers of input cells (indicated below image) are shown. (**F**) Quantification of 1 well representative for 2 – 3 technical replicates with different number of input cells.

First, we sought to verify the specificity and sensitivity of the flow cytometry-based detection assay here presented. For this purpose, PBMC samples were stained for common leukocyte markers, together with two preparations of fluorescently-labeled KLH using either FITC or ReadiLink 700/713. KLH-specific B cells were defined as double positive cells (KLH^++^) within the CD19^+^ CD3^−^CD14^−^CD16^−^CD56^−^ population (Fig. 1a). We employed fluorescence-activated cell sorting to isolate live CD19^+^ KLH^++^ and CD19^+^ KLH^−−^ B cells from patients 5 and 9 during their 3^rd^ cycle of vaccination. Following sorting, cells were stimulated *in vitro* for six days to differentiate them into antibody secreting cells (ASCs). ELISPOT was then used to determine the total number of Ig-secreting ASCs as well as the number of KLH-specific B cells. Although both CD19^+^ KLH^++^ and CD19^+^ KLH^−−^ B cells secreted detectable amounts of total IgG antibodies after *in vitro* differentiation (Fig. 1b), only the CD19^+^ KLH^++^ cells produced KLH-specific antibodies. Enumeration of duplicate wells of CD19^+^ KLH^++^ B cells in patient 9 showed that nearly all IgG-secreting B cells were specific for KLH (Fig. 1c), suggesting a high specificity of the assay. Similar findings were observed for IgM and IgA (Fig. 1d–f).

**Figure 2:**
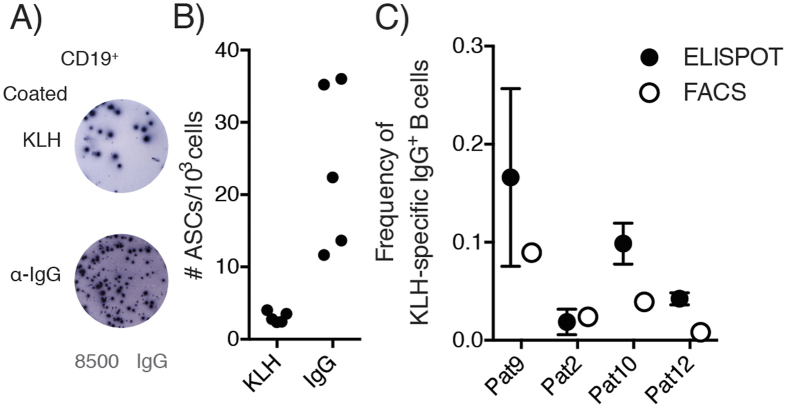
Frequencies of KLH-specific B cells as determined by flow-cytometric analysis are confirmed by ELISPOT analysis. CD19^+^ B cells from patients 2, 9, 10 and 12 after the 3^rd^ cycle of vaccination were sorted and differentiated as described. (**A**) Anti-KLH IgG and total IgG antibody secreting cells were detected using ELISPOT and (**B**) the relative ASC number of both fractions was determined. (**C**) From this data the KLH-specific B cell frequency within the IgG^+^ subset was determined and compared to the frequency of KLH-specific B cells as determined by flow cytometric data at the time of sorting, i.e. before differentiation. Error bars show standard deviation of 2–4 replicate differentiation cultures.

To determine the frequency of circulating KLH-specific B cells via ELISPOT, total CD19^+^ B cells were sorted, differentiated into ASCs and seeded on ELISPOT plates either coated with KLH protein or anti-IgG antibodies (Fig. 2a). Although some variation was observed in the number of IgG-secreting cells per 1000 seeded cells (n = 5 technical replicates, representative for 4 samples), the frequency of anti-KLH IgG antibody producing cells per 1000 cells remained constant (Fig. 2b). To estimate the frequency of KLH-specific IgG^+^ B cells within the sorted B cell population, we determined the ratio of KLH IgG : total IgG ASCs (Fig. 2c). The corresponding ratio was lower yet similar to the frequency of KLH^++^ CD19^+^ IgG^+^ cells, as determined by flow cytometry, suggesting that flow cytometry has a similar sensitivity to ELISPOT.

### Frequency of circulating KLH-specific B cells correlates with anti-KLH antibody serum titers

**Figure 3:**
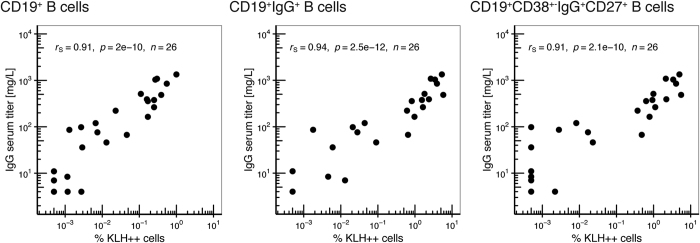
KLH-specific B cell frequencies correlate with anti-KLH IgG antibody titers in serum. Serum titers of anti-KLH IgG antibodies were determined using ELISA and plotted against the frequency of KLH^++^ cells in the indicated B cell subsets. Linear curves were fitted on the data and key regression values are given in each plot.

Next, we correlated the frequency of KLH^++^ cells as determined by flow cytometry with serum levels of anti-KLH antibody titers (Fig. 3). Spearman’s rank-order correlation analysis revealed a strong, positive correlation between the frequency of CD19^+^ KLH^++^ cells and anti-KLH IgG serum titers (r_S_ = 0.91, p = 2·10^−10^, n = 26). This correlation was further improved when comparing serum titers with the frequency of KLH^++^ cells within the CD19^+^ IgG^+^ subpopulation but not the CD19^+^ CD38^+−^IgG^+^ CD27^+^ subpopulation, which has previously been associated with high levels of somatic hyper mutation and a high degree of proliferative potential^21^. Similar correlations, though to a weaker extent, could be found for anti-KLH IgA serum titers and anti-KLH IgM serum titers (Supplementary Figure 1).

### KLH-specific B cell dynamics and phenotype

**Figure 4:**
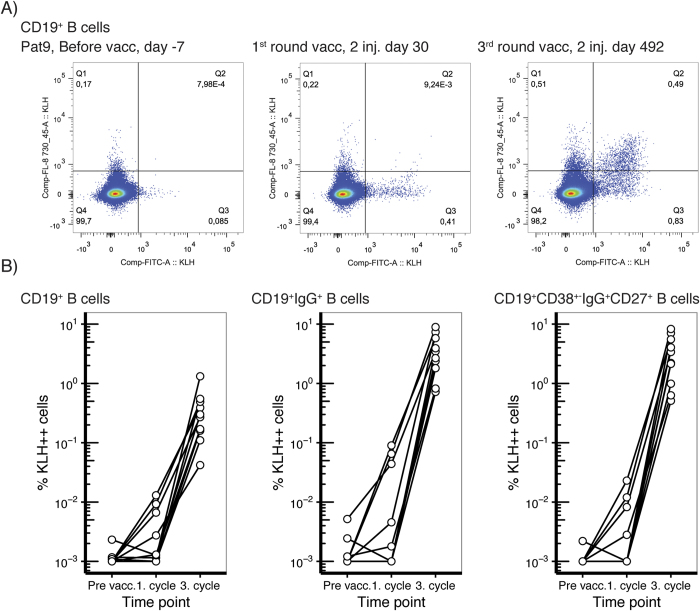
KLH-specific B cells were induced in all vaccinated melanoma patients. KLH-specific B cells were measured before vaccination, after the first cycle and after the third cycle. Days indicate the time to 1^st^ vaccination. (**A**) Dot plots of KLH-specific B cells of one representative melanoma patient. (**B**) Frequencies of the KLH-specific B cells within the indicated B cell subsets for 10 melanoma patients.

Next, we assessed to what extent KLH exposure induced expansion of KLH-specific B cells in our patient cohort (Fig. 4). The baseline frequency of KLH^++^ CD19^+^ B cells was low and did not exceed 0.005% of CD19^+^ B cells. After the 1^st^ cycle, we detected a moderate ~10-fold increase in KLH^++^ B cells in a subset of 3 patients (Fig. 4a,b). After the 3^rd^ cycle, a massive >100-fold expansion was observed in all patients analyzed, reaching frequencies of KLH-specific B cells as high as 1.3% of the total B cell pool (CD19^+^) and 8.9% of the class switched memory B cells (CD19^+^ IgG^+^).

**Figure 5:**
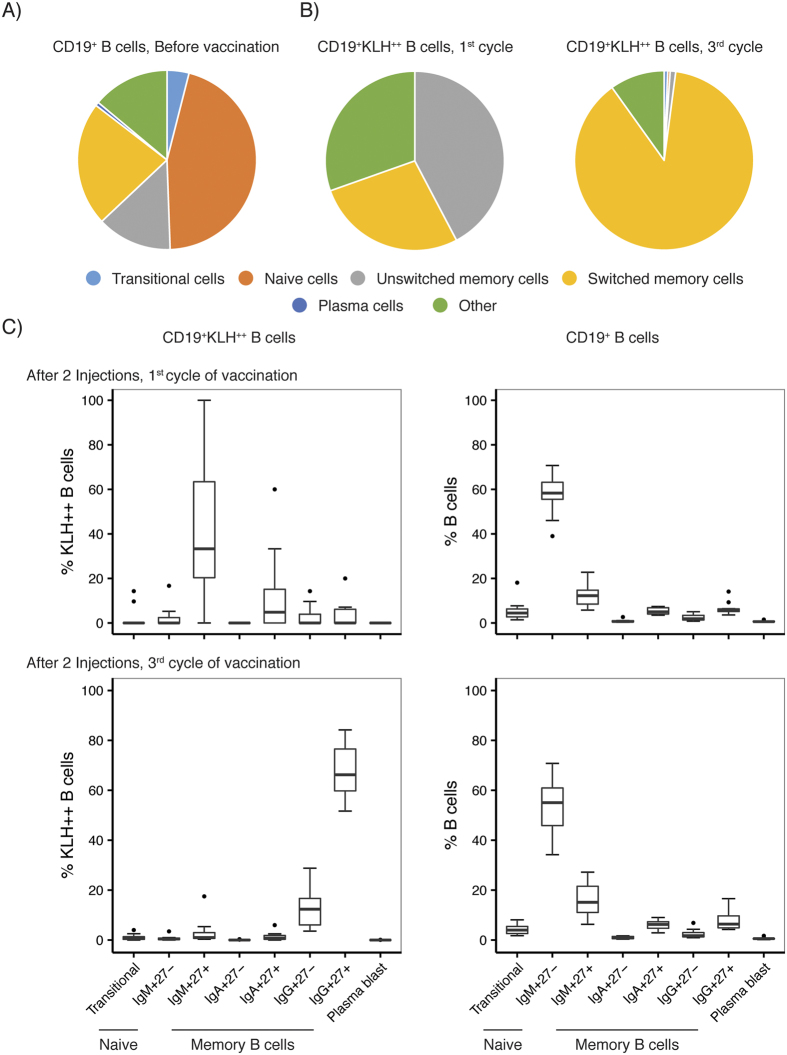
Maturation of KLH-specific B cells during repeated DC vaccination. (**A**) Proportion of different B cell subsets of the total B cells before vaccination (Median values of all patients). (**B**) Proportion of KLH-specific B cell subset after the 1^st^ and 3^rd^ cycle of vaccination. (**C**) In-depth analysis of the development of different B cell subsets within KLH-specific and total B cell population after 1^st^ and 3^rd^ cycle of vaccination (n = 10). Whisker plots represent median (line), 25% and 75%ile (box) and the minimum and maximum values (whiskers). For gating strategy see Supplementary Figure 2.

To further investigate the phenotype of the KLH-specific B cells, we compared changes in expression of cell maturation markers after 1 and 3 cycles of vaccination (Fig. 5). B cell subsets were defined as described (Supplementary Figure 2, Supplementary M&M). After the 1^st^ cycle, CD19^+^ KLH^++^ were predominantly unswitched IgM^+^ CD27^+^ cells. Conversely, after the 3^rd^ cycle, the vast majority of CD19^+^ KLH^++^ cells were class switched B cells and predominantly displayed a IgG^+^ CD27^+^ phenotype, indicative for repeated antigen exposure and late memory differentiation. These maturation changes were specifically observed in the KLH-specific B cell fraction and not in the total CD19^+^ B cell population, which was dominated by IgM^+^ CD27^−^ naïve B cells throughout the entire treatment period, suggesting that these changes specifically occur in KLH-specific B cells.

**Figure 6:**
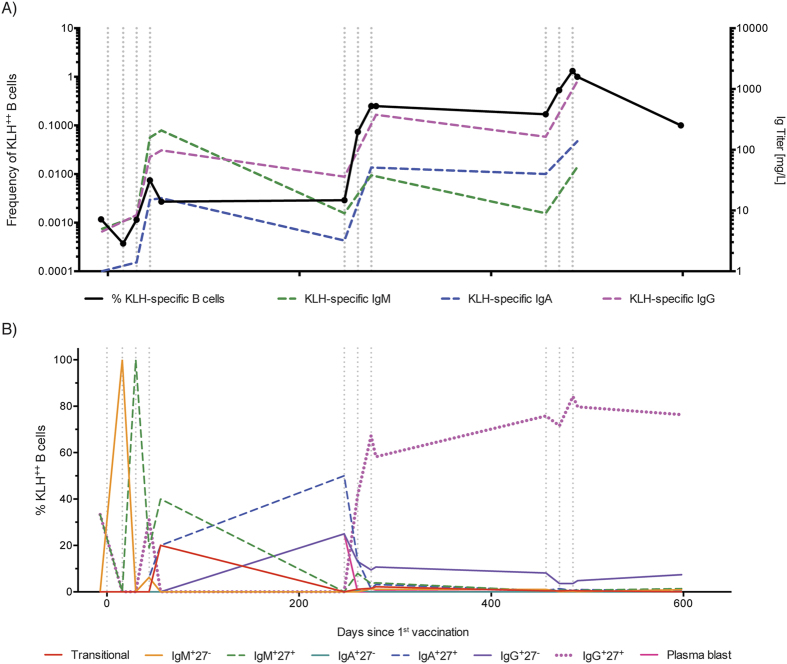
Dynamics of the KLH-specific B cell response during the course repeated KLH vaccinations in patient 5. (**A**) The frequency of KLH^++^ B cells and the serum titer of anti-KLH antibodies at different time points during the treatment of patient 5 are shown. (**B**) The distribution of the KLH^++^ B cells over the different B cell subsets during the course of patient 5′s treatment is shown.

Finally, we analyzed the dynamics of KLH-specific B cell development during the complete course of DC vaccination in patient 5 (Fig. 6). Increased frequencies of KLH^++^ B cells could already be detected after three injections of DCs, in accordance with an increase in anti-KLH Ig titers in serum (Fig. 6a). However, the strongest increase in KLH^++^ B cells was observed after the first vaccination during the 2^nd^ and 3^rd^ cycle. This increase in KLH-specific B cells was accompanied by an increase in anti-KLH IgG antibody titers. Immunophenotyping of KLH-specific B cells confirmed the dynamic changes observed in the serum titers, demonstrating a classical pattern of primary and secondary B cell response (Fig. 6b). Initially, the KLH-specific B cell response was dominated by a subset of IgM^+^ B cells. However, towards the end of the 1^st^ cycle, initial class-switching events were observed with small but detectable increase in IgG^+^ and IgA^+^ cells. During the 2^nd^ cycle, we observed a major expansion of IgG^+^ CD27^+^ memory B cells, and this subset remained the dominant population of KLH-specific B cells up until after the end of the follow-up period of the study.

## Discussion

KLH is an immunogenic agent with various clinical applications and in-depth knowledge on the dynamics of KLH-induced immune responses could aid the understanding of KLH treatment effects.

In this project, we developed a sensitive assay for the *ex vivo* detection of KLH-specific B cells on the basis of the B cell receptor specificity for its cognate antigen and without requirement for B cell pre-stimulation. Using fluorescently labeled KLH, we were able to detect KLH-specific B cells after vaccination of stage III melanoma patients with *ex vivo* KLH-loaded dendritic cells and to phenotype those cells. To verify the specificity and sensitivity, we compared our immunoassay to the current gold standard for the detection of antigen-specific memory B cells, i.e. memory B cell ELISPOT. ELISPOT analysis of sorted KLH^++^ B cells revealed that the great majority of KLH-stained cells was indeed secreting KLH-specific antibodies, demonstrating the high specificity of the assay. To assess the sensitivity, we analyzed the KLH-specific antibody production of total CD19^+^ B cells and observed similar frequencies for both flow cytometry and ELISPOT, suggesting a comparable sensitivity. It should be noted that the frequency of KLH-specific IgG^+^ B cells as measured by flow cytometry was at the lower end of the frequency range as compared to the numbers found by ELISPOT. A potential explanation is that ELISPOT analysis involves an *in vitro* pre-activation step that might introduce bias. The different B cell subsets may not respond to the pre-stimulation conditions in the same manner and thus may not expand at a similar rate as shown, for instance, by Scholzen *et al*. using a similar protocol^22^. On the other hand, our dual staining approach may lead to an under-representation of the actual KLH-specific B cells. Finally, we compared the frequency of KLH-specific B cells with serum anti-KLH antibody titers measured by ELISA. We observed a strong, positive correlation between anti-KLH IgG antibodies and the overall frequency of KLH-specific CD19^+^ IgG^+^ B cells and weak correlation between the frequency of KLH-specific B cells and anti-KLH IgA antibodies or anti-KLH IgM serum titers. Overall, these experiments suggest that the detection of KLH-specific B cells using fluorescently-labeled KLH is feasible, scalable, and in good agreement with established experimental techniques. The advantage of the here described flow cytometric analysis of antigen-specific B cells over ELISPOT analysis is the possibility to combine quantification with extensive phenotyping and purification of cell subsets.

In this study, we found KLH-specific B cell frequencies of up to 1.32% of the total CD19^+^ B cell pool and of up to 8.93% in the CD19^+^ IgG^+^ B cell pool after 3 cycles of DC vaccination. Even 3 months after the last vaccination, KLH-specific B cell frequencies remained as high as 0.1% for CD19^+^ B cells and 0.85% for IgG^+^ CD19^+^ B cells (data not shown). To put this in perspective, we compared our results with other published studies on flow cytometry-based detection of antigen-specific B cells. In healthy volunteers undergoing Tetanus toxoid (TT) booster vaccination, for instance, numbers of TT-specific CD19^+^ B cells peaked at 0.012%; two orders of magnitude lower than the numbers of KLH-specific CD19^+^ B cells we observed^17^. Of note, the time points after booster vaccination may differ between the cited study and ours as exact details are only given for a subset of TT-vaccinated volunteers. Other studies examined naturally induced B cell responses, for instance in allergic patients under oral immunotherapy. Here, up to 0.3% peanut allergen Arah1– and Arah2-specific CD19^+^ B cells were detected^18^. Kerkman *et al*. observed up to 0.06% of CD19^+^ B cells being specific for a mimic of citrullinated protein antigens^20^. When examining the IgG-specific B cell response in viremic HIV patients, Kardava *et al*. discovered up to 0.3% of Tetanus- or influenza-specific CD19^+^ IgG^+^ B cells^19^. In the same study, the authors could detect up to 6% of CD19^+^ IgG^+^ B cells being specific for the HIV antigen gp140. Our results thus confirm the high immunogenicity of KLH but also show that direct comparison of specific B cell numbers are difficult between studies due to differences in timing and activation state of the immune system.

The KLH-specific B cell maturation patterns we observed in this study closely follow prototypic primary and secondary immune responses against a neo-antigen. The primary response was dominated by KLH^++^ IgM^+^ B cells, which switched to an IgG-dominated response after secondary and tertiary immunization, aligning closely with earlier studies on anti-KLH serum antibody levels^23^,^24^. Moreover, early KLH-specific IgG^+^ B cells were predominantly CD27^−^, whereas late KLH-specific IgG^+^ B cells were CD27^+^. This is in line with the concept that IgG^+^ CD27^−^ B cells underwent one germinal center reaction and become CD27^+^ upon subsequent antigen exposure and additional germinal center reactions^21^. We also detected relatively high levels of IgA^+^ KLH-specific B cells in early responses, which were not detected at later time points. Although it remains unclear whether this B cell subset contracts after subsequent antigen exposure or whether these cells home to other (mucosal) tissues, the observed increase in anti-KLH IgA antibody titers in patients after multiple vaccinations argues for the latter option. Our data collectively indicate that the employed DC vaccination is inducing an IgG-dominated humoral immune response possibly driven by the induction of Th1 cells typically observed during this treatment^25^. The question remains as to how far the induction of KLH-specific B cells early after vaccination can be predictive for the clinical response to dendritic cell-based vaccination. Further research in extended cohorts is necessary to investigate this.

Although it is generally assumed that the primary activation signal of B cells is direct contact of the B cell receptor with its cognate antigen, it should be noted that the patients in our study were never vaccinated directly with the soluble KLH antigen. Instead, patients were injected with autologous *ex vivo* differentiated monocyte-derived dendritic cells. Following differentiation, DCs were incubated with soluble KLH, after which cells were washed thoroughly. Although this procedure efficiently removes soluble KLH, we still observe robust humoral KLH-responses. The mechanism by which KLH is able to induce a B cell response in these patients needs thus further investigation.

In summary, this study shows a novel, fast, and robust method to quantify and phenotype circulating KLH-specific B cells. The use of flow cytometry does not only allow more in-depth phenotyping of KLH-specific B cells, but also allows isolation, functional analysis, molecular profiling, tracking of clonal evolution and production of fully human antibodies. By further standardization, the flow cytometric assay presented here may allow for inter-institutional comparisons and immunomonitoring of KLH-specific B cell responses. This will help to increase our understanding of the human B cell response to KLH and further facilitate the development of novel clinical KLH applications.

## Methods

### Patients

Peripheral blood mononuclear cells (PBMCs) or peripheral blood lymphocytes (PBLs) were collected from stage III melanoma patients at different time points during the course of their disease (n = 10). All patients underwent immunotherapy using autologous monocyte-derived matured dendritic cells loaded with KLH and tumor antigen in our department as described in the original study and indicated in Supplementary Table 1^26^. Patients received DCs in three cycles every 6–9 months, with three fortnightly injections per cycle, except for the 1st cycle where four injections were given. Patients were World Health Organization performance status 0 or 1^27^. The original study (Clinical trial registration number NCT00243594, registration date: 21.10.2005) was in accordance with relevant guidelines and regulations and approved by the Medical Research Ethics Committee (CMO Regio Arnhem-Nijmegen). Patients signed informed consent.

### KLH-labeling

KLH protein (Vacmune, Immucothel) was labeled with either FITC or Readilink 700/713 using the FITC Antibody Labeling Kit (Pierce, 53027) or the ReadiLink 700/713 Antibody Labeling Kit (BioRad, 135-1008), respectively, following the manufacturer’s instructions.

### B cell staining

Cryopreserved PBMCs and PBLs were thawed and washed 2x in Iscove’s Modified Dulbecco’s Medium (IMDM) (Gibco) supplemented with 1% fetal bovine serum (FBS) (Greiner Bio-One) and 25 μg/mL DNAse 1 (Roche). The serum lot was pretested for performance. In case of several time points measured for a single patient, all samples were processed in one experiment, with the exception of patient 5. Subsequently, cells were washed 2x with PBS and dead cells were identified by staining with Fixable Viability Dye eFluor^®^ 780 (eBioscience, 1:2000 in PBS, 100 μL) at 4 °C for 30 minutes. Cells were washed once with PBS followed by one wash with PBS supplemented with 2% FBS (wash buffer). B cells (5–50 · 10^6^) were stained with 10 μg/mL FITC- and 1 μg/mL ReadiLink 700/713-labeled KLH in 120 μL wash buffer for 30 minutes on ice. Cells were pulse vortexed every 5 minutes during incubation. Subsequently, cells were incubated for 20 minutes in a total volume of 200 μL of fluorescently labeled antibodies in wash buffer. See Supplementary Methods for the labeled monoclonal antibodies used in this study. Cells were washed 1x, resuspended in wash buffer and kept at 4 °C for a maximum of 2 h before analysis by flow cytometry.

### Flow cytometry and gating strategy

Cells were acquired using a LSRII and – for fluorescence-activated cell sorting – a FACSAria (both Becton Dickinson) and analyzed with FlowJo software (Tree Star). Per sample, between 867,000 and 16,600,000 events were acquired, yielding 38,720–632,000 viable CD19^+^ B cells. The lymphocyte population was gated followed by exclusion of doublets and dead cells. B cells were defined as CD19^+^ CD3^−^CD14^−^CD16^−^CD56^−^ and KLH-specific B cells identified by dual staining for FITC and ReadiLink 700/713 (Fig. 1). During the 1^st^ vaccination cycle, the frequencies of KLH-specific B cells were relatively low and calculations were based on up to 30 KLH-specific B cells. B cell subsets were characterized using a gating strategy adapted from Berkowska *et al*. (Supplementary Figure 2 and Supplementary Methods)^21^.

### B cell expansion and ELISPOT

Sorted B cells were quantified and checked for antibody production using ELISPOT. See Supplementary Methods for a detailed description of the B cell expansion protocol and ELISPOT procedure.

### KLH-specific antibody production

The concentration of KLH-specific antibodies was determined by ELISA (www.KLHanalysis.com) in patients’ sera before and at various time points after vaccination. Serum samples were obtained at a maximum of +/− 10 days before/after the corresponding PBMC samples. See Supplementary Methods for a detailed description of the ELISA procedure.

### Data Analysis and Statistics

Analysis and presentation of distributions was performed using PRISM for windows version 5.03 (GraphPad) and The R Project for Statistical Computing using the ggplot2 and xlsx packages^28^,^29^,^30^. For statistical analysis, Spearman’s Rank-Order correlation analysis was employed.

## Additional Information

**How to cite this article:** Wimmers, F. *et al*. Monitoring of dynamic changes in Keyhole Limpet Hemocyanin (KLH)-specific B cells in KLH-vaccinated cancer patients. *Sci. Rep.* **7**, 43486; doi: 10.1038/srep43486 (2017).

**Publisher's note:** Springer Nature remains neutral with regard to jurisdictional claims in published maps and institutional affiliations.

## Supplementary Material

Supplementary Information
