# Supplementary material for: Monitoring of dynamic changes in Keyhole Limpet Hemocyanin (KLH)-specific B cells in KLH-vaccinated cancer patients
Source: Sci Rep. 2017 Mar 7;7:43486. doi: 10.1038/srep43486 (PMC5361210; doi:10.1038/srep43486)
Supplement: Supplementary Information [file srep43486-s1.pdf]

## Supplementary Information

# Monitoring of dynamic changes in Keyhole Limpet Hemocyanin (KLH)-specific B cells in KLH-vaccinated cancer patients

Florian Wimmers, Nienke de Haas, Anja Scholzen, Gerty Schreibelt, Elles Simonetti, Marc J. Eleveld, Huberdina M.L.M. Brouwers, Marjo Beldhuis-Valkis, Irma Joosten, Marien I. de Jonge, Winald R. Gerritsen, I. Jolanda M. de Vries*, Dimitri A. Diavatopoulos*, Joannes F.M. Jacobs

* Authors contributed equally

# Supplementary Data

**Supplementary Figure 1 – Correlation of KLH-specific B cell frequencies and anti-KLH IgA and IgM antibodies.** Serum titers of anti-KLH IgM (A) and IgA (B) antibodies were assessed via ELISA and plotted against the frequency of KLH$^{++}$ cells in the indicated B cell subsets. Linear curves were fitted on the data and key regression values are indicated in each plot.

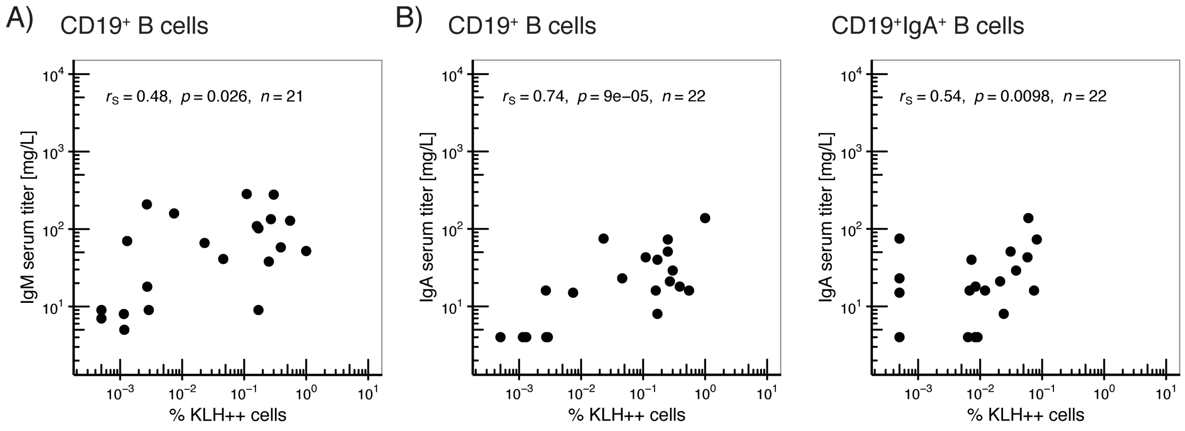

**Supplementary Figure 2– Gating scheme for identification of KLH-specific B cells and memory subsets.** PBMCs of melanoma patients were thawed, washed and stained using a FITC- and ReadiLink700/713-labeled KLH as well as a cocktail of fluorescently-labeled antibodies. KLH-specific B cells and various B cell subsets were identified using the illustrated gating scheme.

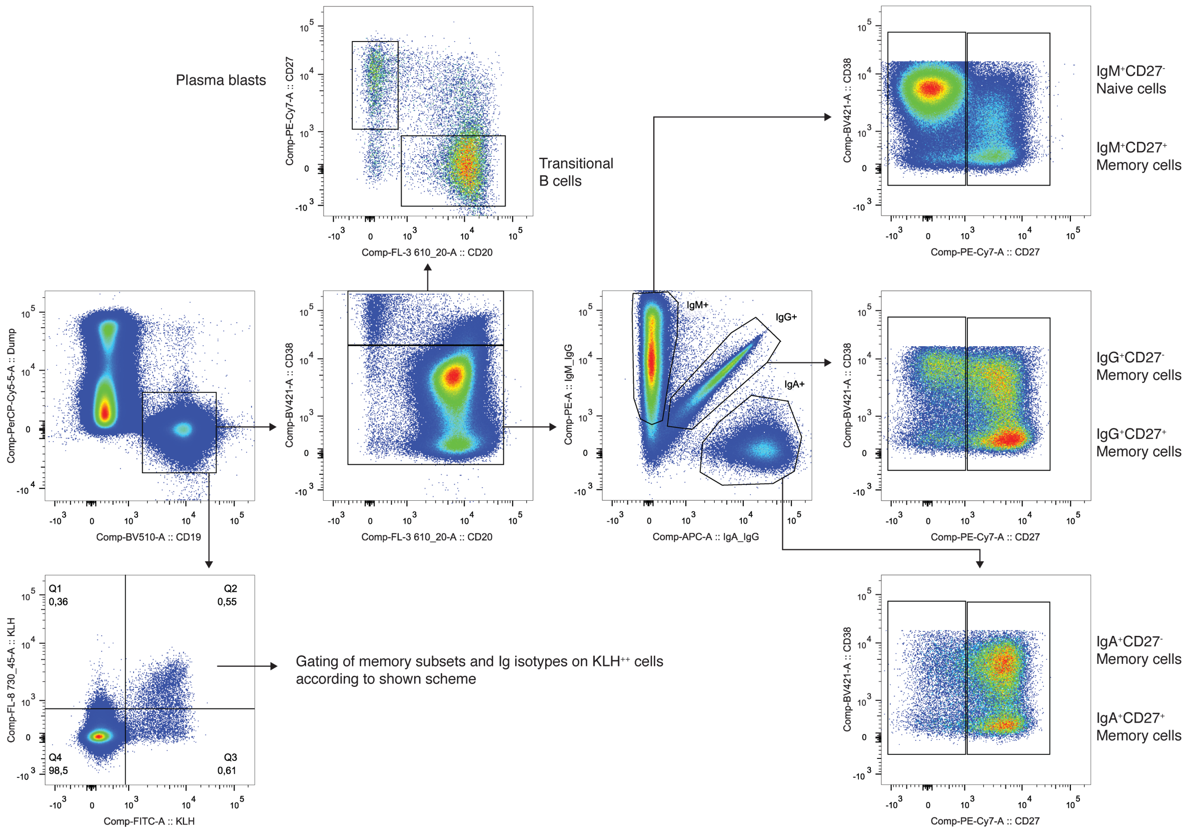

**Supplementary Table 1 - Patient characteristics**

| | | | | | | | Vaccination parameters | | |
|---|---|---|---|---|---|---|---|---|---|
| Patient | Age | Sex | N stage baseline | AJCC stage baseline | RFS (mo) | OS (mo) | Route | IL-2 | Cycles |
| Pat 1 | 51 | F | N1a | IIIA | 80.8+ | 80.8+ | i.d. | Yes | 3 |
| Pat 2 | 35 | M | N1b | IIIC | 121.9+ | 121.9+ | i.n. | Yes | 3 |
| Pat 5 | 64 | M | N1a | IIIA | 80.4+ | 80.4+ | i.d. | Yes | 3 |
| Pat 6 | 59 | M | N3 | IIIC | 63.1 | 80.6 | i.d. | No | 3 |
| Pat 7 | 39 | M | N1b | IIIB | 133.0+ | 133.0+ | i.n. | Yes | 3 |
| Pat 8 | 59 | M | N3 | IIIC | 15.9 | 21.4 | i.d. | Yes | 3 |
| Pat 9 | 48 | M | N3 | IIIC | 10.8 | 96.7+ | i.d. | No | 3 |
| Pat 10 | 64 | F | N3 | IIIC | 140.8+ | 140.8+ | i.d. | Yes | 3 |
| Pat 11 | 38 | F | N1b | IIIC | 43.1 | 59.0 | i.d. | Yes | 3 |
| Pat 12 | 44 | F | N1a | IIIA | 165.0+ | 165.0+ | i.d. | Yes | 3 |

Abbreviations: i.n., intranodal; i.d., intradermal; AJCC, American Joint Committee on Cancer; RFS, recurrence-free survival; OS, overall survival.
[a]Number of received vaccination cycles [1 cycle consists of 4 (first cycle) or 3 (second and third cycle) vaccinations and a DTH test].

# Supplementary Methods

## Patients

Peripheral blood mononuclear cells (PBMCs) or peripheral blood lymphocytes (PBLs) were collected from melanoma patients with loco regional resectable disease (stage III), scheduled for regional lymph node dissection at different time points during the course of their disease (n=10). All patients underwent immunotherapy using autologous monocyte-derived matured dendritic cells loaded with KLH and tumor antigen in our department as described in the original study and indicated in Supplementary Table 1 (1). Briefly, patients underwent leukapheresis and monocytes were isolated and within 5 - 8 days were differentiated into monocyte-derived DCs using IL-4 and GM-CSF. KLH is added to this culture at day 4 after isolation. Subsequently, DCs were activated using monocyte-conditioned medium enriched with prostaglandin $E_2$ and TNF$\alpha$, and loaded with cancer antigens by pulsing with gp100- or tyrosinase-derived peptides. Finally, the prepared DC vaccine was administered to patients intradermally or intranodally at 12 - $17\times10^6$ dendritic cells per injection. All patients received DCs in three cycles, with three fortnightly injections per cycle, except for the 1st cycle where four injections were given. A subset of the included patients also received subcutaneous injections of interleukin-2 (IL-2) (at 9 MIU) once daily for one week starting at three days after each DC vaccination (Supplementary Table 1). Patients were confirmed to be HLA*02:01 and World Health Organization performance status 0 or 1 and primary tumors or metastases were tested for tumor-associated antigen expression (gp100 and tyrosinase) (2). The original study (Clinical trial registration number NCT00243594, registration date: 21.10.2005) was approved by the Medical Research Ethics Committee (CMO Regio Arnhem-Nijmegen) and patients signed informed consent.

**Sample acquisition**

Whole blood was obtained by venipuncture using sodium heparin-containing vacutainer collection tubes or leukapheresis. PBMCs were isolated via gradient centrifugation using Lymphoprep (Axis-Shield) following the manufacturer's instructions. PBLs were obtained by depleting monocytes via plastic adherence. Cells were frozen in X-VIVO 15 (Lonza) supplemented with 40% Albuman 200g/L (Sanquin) and 10% CryoSure-DMSO (WAK Chemie Medical GMBH) at 10–100×$10^6$ cells per vial using Cryo 1°C Freezing Container (NALGENE) following the manufacturer's instructions and transferred into the vapor phase of liquid nitrogen after 72 hours for long-term storage.

**Monoclonal antibodies**

For staining of B cells the following monoclonal antibodies were employed: BV510-labeled anti-CD19 (SJ25C1, 1:50), PE-CF594-labeled anti-CD20 (2H7, 1:400) and BV421-labeled anti-CD38 (HIT2, 1:20, all BD Horizon), PE-Cy7-lableld anti-CD27 (1A4CD27, 1:12) and PE-labeled anti-IgM (SA-DA4, 1:100, both Beckman Coulter), PE-labeled anti-IgG (G18-145, BD 1:25) and APC-labeled anti-IgG (G18-145, 1:25, both BD Pharmingen), APC-labeled anti-IgA (IS11-8E10, Miltenyi, 1:100), PerCP-Cy5.5-labeled anti-CD3 (SK7, 1:400, eBioscience) and anti-CD14 (M5E2, 1:100), anti-CD16 (3G8, 1:100) and anti-CD56 (HCD56, 1:80, all Biolegend). Cells were washed 1x, resuspended in wash buffer and kept at 4°C for a maximum of 2h before analysis by flow cytometry.

**Flow cytometry and Gating strategy**

B cell subsets were characterized using a gating strategy adapted from Berkowska et *al.* (Supplementary Figure 1) (3). Briefly, naïve B cells were defined as $CD38^{+/-}IgM^{+}CD27^{-}$,

unswitched memory B cells were defined as $CD38^{+/-}IgM^{+}CD27^{+}$, and class-switched memory B cells as $CD38^{+/-}IgG^{+}CD27^{-/+}$ or $CD38^{+/-}IgA^{+}CD27^{-/+}$. Transitional cells were identified by the expression of $CD38^{++}CD27^{-}CD20^{+}$ and plasma blasts were defined as $CD38^{++}CD27^{+}CD20^{-}$.

**B cell expansion and ELISPOT**

Sorted B cells were counted and resuspended in 200 µL IMDM containing 10% FBS, 1% antibiotic-antimycotic (Gibco) (complete IMDM) supplemented with 10 ng/mL IL-21 (eBioscience), 40 U/mL IL-2 (PROLEUKIN® $18 \cdot 10^{6}$ IU, Novartis), 10 ng/mL IL-10 (Immunotools), 1 µg/mL ODN-2006 CpG (Enzo Lifesciences) and $5 \cdot 10^{3}$ CD40 ligand-transfected mouse fibroblasts. Mouse fibroblasts were cultured in complete IMDM with 1 mg/mL G418 Geneticine (Gibco/Invitrogen). Before coculture with B cells, fibroblasts were detached by treatment with 0.5 mg/mL trypsin (Difco) for 5 minutes at 37°C, collected, washed in complete IMDM, irradiated at 60 Gy and added to the stimulation mix. B cells were cultured in 96-well round bottom plates (Costar, polystyrene) for 6 days and medium including stimulation mix was refreshed after 3 days.

MultiScreen Filter PVDF Immobilon plates (Millipore) were pre-wetted with 30 µL/well of a 35% ethanol solution. Immediately after pre-wetting, plates were washed 2x with PBS. Plates were coated over night at 4°C with 50 µL PBS containing 10 µg/mL of anti-IgG (MT91/145, Mabtech) or KLH protein, washed with PBS containing 0.05% Tween 20 (PBST) (Sigma-Aldrich) and 3x PBS. Subsequently, plates were blocked with 100 µL complete IMDM for 2h at 37°C. B cells were washed in complete IMDM and – in case of sorted total $CD19^{+}$ cells – counted. Cells were resuspended in 200 µL complete IMDM at the desired concentration. 100 µL cell suspension was added to each well coated with KLH or anti-IgG and incubated for 6h at 37°C. Subsequently, plates were washed 4x with PBS and 4x with PBST and incubated over night at

4°C with 50 µL/well PBST containing 0.5% FBS and alkaline phosphatase-labeled polyclonal goat anti-human IgG (Fcγ) antibodies (1:1000, Mabtech). Plates were washed with 4x PBST, 4x PBS and 3x deionized water. All washing steps were performed with 200 µL/well. Subsequently, plates were incubated with 50 µL/well 0.45 µm-filtered alkaline phosphatase substrate BCIP/NBT (Mabtech) and kept in the dark until fully developed. Finally, plates were rinsed with deionized water and protected from light until scanned and analyzed using ELI.Analyse software (A.EL.VIS).

**KLH-specific antibody production**

Microtiter plates (96 wells) were coated overnight at 4°C with 120 µL/well KLH (25 µg/mL in PBS). Wells were subsequently incubated for 1 hour at room temperature with milk powder blocking buffer (1% in 0.01 M PBS), 200 µL/well. Plates were washed in an automatic washer (Tecan). Each wash step in this protocol includes two automated wash-cycles using 300 µL/well 0.01 M PBS. Patient serum was diluted in two concentrations (50 and 150 times diluted) in 0.01 M PBS and 100 µL/well was added in duplicate for 1 hour at room temperature. After washing, patient KLH-specific antibodies were detected with monoclonal mouse anti-human IgG (Invitrogen, clone HP6017, diluted 1000 times in 1% PBA), IgM (Invitrogen, clone HP6083, diluted 1500 times in 1% PBA) antibodies, or polyclonal goat anti-human IgA (Cappel, diluted 3000 times in 1% PBA) that are labeled with horseradish peroxidase. After 1 hour of incubation, plates were washed and 100 µL/well 3,3′ 5,5-tetramethyl-benzidine was added as a substrate (Dako). After five minutes the reaction was stopped with 100 µL/well 1M sulfuric acid and plates were measured in a microtiter plate reader (Tecan) at 450 nm. An isotype-specific calibration curve for the KLH response was included on each microtiter plate (4).

**General Lab Operation**

These studies were conducted in a laboratory that operates under exploratory research principles. The study was performed using established laboratory protocols.

## *References*


1. Lesterhuis, W. J. *et al*. Route of administration modulates the induction of dendritic cell vaccine-induced antigen-specific t cells in advanced melanoma patients. *Clin Cancer Res* **17**, 5725 - 35 (2011).
2. Oken, M. M. *et al*. Toxicity and response criteria of the eastern cooperative oncology group. *Am J Clin Oncol* **5**, 649 – 55 (1982).
3. Berkowska, M. A. *et a*l. Human memory b cells originate from three distinct germinal center-dependent and -independent maturation pathways. *Blood* **118**, 2150 – 8 (2011).
4. Aarntzen, E. H. *et al*. Humoral anti-klh responses in cancer patients treated with dendritic cell-based immunotherapy are dictated by different vaccination parameters. *Cancer Immunol Immunother* **61**, 2003 – 11 (2012).
